# Green analytical chemistry: integrating sustainability into undergraduate education

**DOI:** 10.1007/s00216-024-05680-4

**Published:** 2024-12-06

**Authors:** Saša M. Miladinović

**Affiliations:** School of Engineering, Institute of Life Sciences, HES-SO University of Applied Sciences Western Switzerland, Rue de L’Industrie 19, 1950 Sion, Switzerland

## Introduction

Green chemistry has become a fundamental aspect of modern scientific research and industrial practice, promoting the development of sustainable technologies that minimize environmental impact and improve safety for both humans and ecosystems. Integrating green chemistry principles into various branches of chemistry, including analytical chemistry, underscores the importance of sustainability in all chemical processes [1].

Green analytical chemistry (GAC) is defined as the optimization of analytical processes to ensure they are safe, nontoxic, environmentally friendly, and efficient in their use of materials, energy, and waste generation [2]. The GAC field is guided by 12 principles that prioritize sustainability, such as waste prevention, the use of safer solvents and reaction conditions, and energy efficiency [3–5]. These principles serve as a framework for developing methodologies that are both effective and environmentally friendly.

In an era where sustainability and environmental responsibility are crucial, it is increasingly important for analytical chemists to be familiar with the principles of GAC. This paper describes a green analytical chemistry course developed for bachelor’s students specializing in analytical chemistry during their third and final year of study. As environmental regulations tighten and industries shift towards greener practices, this course equips future chemists with the skills to create methods that are not only efficient but also environmentally friendly. By adopting GAC principles, chemists contribute to environmental protection and gain a competitive edge as industry trends increasingly favor green solutions. This alignment between analytical chemistry and sustainability is essential for addressing the complex challenges of the twenty-first century, balancing scientific progress with ecological preservation.

Traditionally, analytical methods have often relied on toxic reagents and solvents, which can generate significant waste and pose potential risks to both analysts and the environment. GAC addresses these challenges by optimizing analytical processes to be inherently safer and more sustainable [6–8].

Integrating green chemistry into analytical chemistry curricula is essential for several reasons including the following:

1. Environmental responsibility: Analytical chemists should recognize the environmental impact of their work and strive to minimize it through sustainable practices.
2. Safety: The adoption of green principles reduces exposure to hazardous chemicals, creating a safer environment for both students and professionals.
3. Economic efficiency: Green methods can reduce costs by minimizing the consumption of reagents and energy as well as the production of waste.
4. Regulatory compliance: As environmental regulations become more rigorous, knowledge of GAC ensures that future chemists can develop methods that meet these standards.
5. Innovative thinking: Incorporating green chemistry encourages innovative approaches to problem-solving, fostering a mindset that values sustainability alongside scientific excellence.

### Objectives of the course

The primary objective of this course is to introduce undergraduate students enrolled in Bachelor of Science (BSc) in analytical chemistry program to the principles and practices of green analytical chemistry, with a strong emphasis on its application across various scientific and industrial settings. This course will introduce students to the concepts of green chemistry, as they have not previously taken a course dedicated to this subject. By integrating the concept of sustainability into the curriculum, this course aims to foster a way of thinking that prioritizes environmentally responsible approaches in chemical analysis. Students are encouraged to critically evaluate traditional practices and explore innovative methods that reduce the environmental impact of chemical processes. Through this course, students develop a deep understanding of the importance of sustainability in science and industry, equipping them with the skills and mindset necessary to contribute to a more sustainable future.

The course’s specific goals are as follows:

1. Understand the principles of green chemistry: Students gain a comprehensive understanding of the 12 principles of green chemistry and their application in designing safer, more sustainable chemical processes, emphasizing the importance of reducing environmental impact.
2. Evaluate traditional analytical methods: Students critically evaluate traditional analytical techniques, identifying opportunities to enhance sustainability and reduce ecological footprints in chemical analysis.
3. Develop green analytical methods: Students learn how to theoretically design greener analytical methods that reduce environmental impact while also improving safety and efficiency in chemical processes.
4. Promote a sustainable mindset: Through case studies, students develop a mindset that prioritizes sustainability, preparing them to integrate sustainable practices into all aspects of chemical analysis.

This course equips students with the knowledge and skills necessary to pursue careers in chemistry, where they can lead efforts to advance sustainable practices and drive positive change within the scientific community. In addition to the foundational principles of GAC, the course design is supported by active learning pedagogies that highlight student engagement and critical thinking. The jigsaw technique [9], a collaborative learning strategy, was specifically chosen to enhance peer learning and foster a deeper understanding of GAC principles. In this approach, the class is divided into initial three groups of students where students read and discuss certain topic. Following this discussion, in the second part of class, the students are reorganized into three new groups. In the newly formed groups, students explain and share their initial discussions with their new group peers, promoting deeper understanding and collaborative learning. Such approaches align with the broader educational shift towards sustainability education, where the focus is not only on knowledge acquisition but also on developing the skills necessary for students to address complex environmental challenges in their future careers [10].

## Course design and structure

In Switzerland, the Universities of Applied Sciences have a specific role within the higher education system. Their primary purpose is to provide practice-oriented education and training that prepares students directly for careers in specific professional fields. Universities of Applied Sciences primarily attract students who are looking for a direct path into specific careers. Students may prefer practical training over purely academic or theoretical studies. This highlights the essential role of these universities in bridging the gap between academic learning and industry requirements, thereby preparing graduates for seamless integration into the industry.

The development of a curriculum for these institutions, particularly in specialized fields such as green analytical chemistry (GAC), requires careful consideration of both relevant literature and educational resources covering theoretical and practical aspects. In designing a GAC course for final-year bachelor’s students majoring in analytical chemistry, the *Handbook of Green Analytical Chemistry* by de la Guardia and Garrigues [11] was identified as an essential resource. This text was selected not only because it covers the fundamental concepts of GAC but also because it aligns well with the practical and application-focused principles of Universities of Applied Sciences. The curriculum topics were carefully selected to include a variety of analytical techniques, ensuring that students acquired knowledge directly applicable to their future professional careers. Particular emphasis was placed on the “greening” of existing analytical procedures, which students had already encountered and practiced in laboratory settings during the first 2 years of the bachelor’s study.

Table 1 provides an overview of the course structure, detailing the specific learning objectives, key activities, and corresponding assessment methods for each unit.

**Table 1:** Overview of green analytical chemistry course structure

Unit	Learning objectives	Key activities
Unit 1: Concepts of green analytical chemistry	Should be able to describe how the principles of GAC are utilized in designing a chemical analysis method and explain their role in promoting sustainability in chemical analysis	Lecture on GAC principles and comparison with traditional methods Group discussion using the jigsaw technique on selected GAC milestones and development [11]
Unit 2: Analytical method assessment	Should be able to identify and utilize tools to assess the greenness of analytical methods	Introduction to greenness assessment tools Exercise evaluating selected publications using greenness assessment tools Group discussion using the jigsaw technique on tools for assessing the “greenness” of the method [12–14]
Unit 3: The analytical process	Should be able to describe and evaluate green sampling and preparation techniques	Lecture on green sampling techniques Case study analysis and critical reading of research papers on green analytical innovations
Unit 4: Greening strategies in analytical chemistry	Should be able to compare strategies to reduce the carbon footprint and energy consumption in laboratories Should be able to describe the benefits of miniaturization and alternative energy sources	Lecture on energy-saving strategies in the lab Practical exploration of alternative solvents and energy sources
Unit 5: Applications in green analytical chemistry	Should be able to apply GAC principles into the design of bioanalysis and environmental analysis methods	Lecture on green bioanalysis methods Case study analysis of industrial and environmental applications of switchable solvents, conducted through a group jigsaw technique [15]
Unit 6: Student assessment presentation	Should be able to present a comprehensive greenness analysis of a current analytical method and propose greener alternative	Student pairs work on a selected method from the European Pharmacopoeia Group presentation of proposed improvements and reassessment using GAPI/AGREE tools

A total of 12 class hours were allocated for this course, with each instructional unit being covered over the span of 2 class hours. Detailed descriptions of each unit, including topics covered, learning objectives, and relevance to green analytical chemistry, are described below.

*Unit 1: Concepts of green analytical chemistry* introduces students to the foundational concepts of sustainability and the definition of green analytical chemistry (GAC) [8]. The 12 principles of GAC are presented, along with a detailed explanation of the differences between traditional analytical figures of merit and those specific to green analytical practices [16]. The concept of “greening” analytical chemistry is explored as a methodology to make analytical procedures more environmentally sustainable [2]. Additionally, the challenges associated with implementing green practices are discussed [17]. As a practical example, the significance of flow injection analysis within the context of GAC is explained [18].

To reinforce these concepts, students select one milestone from the *Handbook of Green Analytical Chemistry* (from Fig. 1.3 in the textbook [11]) and discuss how this milestone has contributed to advancing green practices in analytical chemistry and explain its practical use. The discussion and critical analysis of the material are facilitated using the jigsaw didactics technique [9]. The class of 12 students is divided into initial 3 groups of students where students discuss the significance of chosen GAC milestone. Following this discussion, in the second part of the class, the students are reorganized into three new groups. In the newly formed groups, students explain and share their initial discussions with their new group peers, explaining why they choose to discuss certain milestone and how it influenced the development of GAC.

**Fig. 1:**
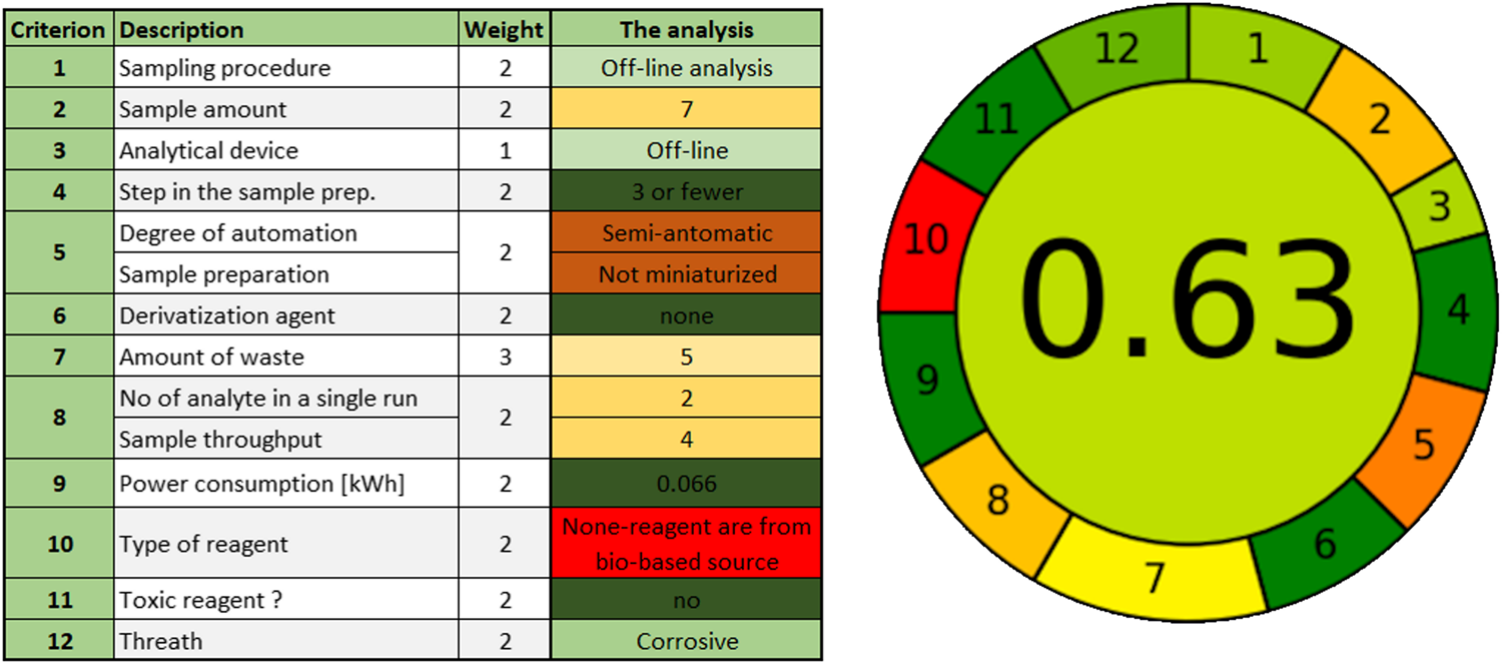
The AGREE tool for greenness assessment

*Unit 2: Analytical method assessment* introduces students to the concept of Analytical Method Volume Intensity (AMVI) as a starting point for evaluating analytical methods. Initial discussion compares two high-performance liquid chromatography (HPLC) methods described by Hartman et al*.* [19]. Various tools for assessing the greenness of analytical methods are introduced starting with the National Environmental Methods Index (NEMI) [20], followed by the Green Analytical Procedure Index (GAPI) [21], and finally, the Analytical GREEnness (AGREE) tool [22]. The application of these three greenness assessment tools are discussed for stability-indicating assays [20]. Students are provided with practical guidance on using the GAPI spreadsheet and AGREE software. These tools are essential for evaluating the environmental impact of analytical methods, particularly in the context of sustainable chemistry. Students were introduced to the NEMI tool but did not practice using it in their method evaluations. Figure 1 illustrates the AGREE tool, which offers a holistic evaluation of the greenness of a method based on 12 distinct criteria. This comprehensive approach helps identify areas of improvement for developing more environmentally friendly analytical procedures.

**Fig. 2:**
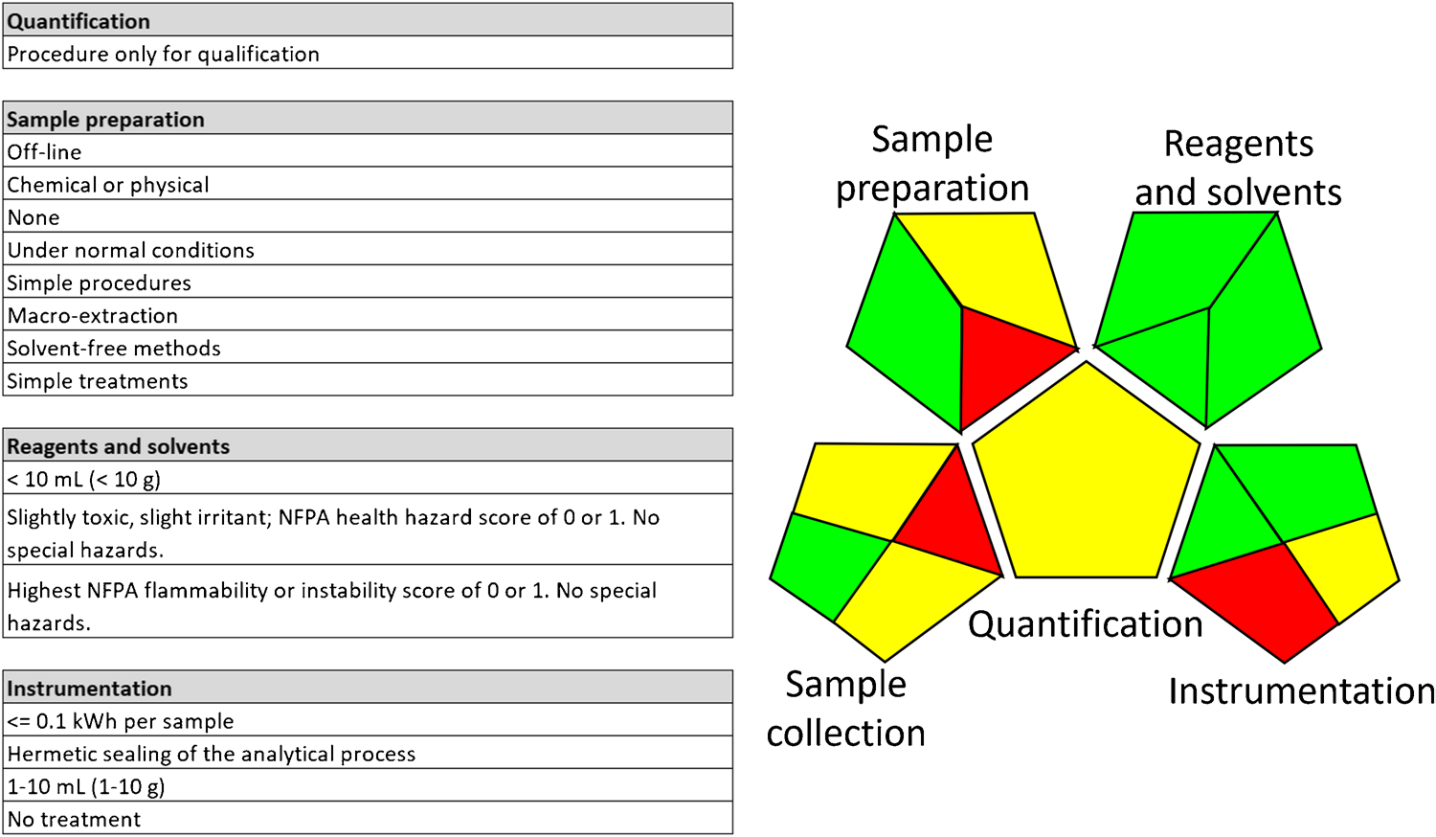
The GAPI tool for greenness evaluation

Figure 2 presents the GAPI tool, which is designed to assess the greenness of an analytical method using a color-coded system that is easy to interpret. The GAPI tool considers the entire life cycle of the method, from reagents and solvents used to waste management, providing a thorough greenness evaluation. The pictures are extracted from students presentation included in the supplementary material.

The second part of the unit is dedicated to a group exercise. The class is divided into three groups, each assigned one of three selected publications [12–14]. Students are tasked with assessing the greenness of one of the methods. The jigsaw technique is again utilized, allowing students to share and compare their assessments within newly formed groups. These publications were carefully chosen for their representation of relatively simple techniques, ensuring that the assessment tasks are manageable for students who had just been introduced to the greenness assessment tools. This structure allows the entire exercise to be completed within the two classes allotted for the unit, without requiring additional work outside of class.

*Unit 3: The analytical process* begins with a comprehensive introduction to the analytical process, emphasizing the integration of green techniques. The unit opens with the introduction of green solvents in the sampling process. Concepts such as Total Hazard Value (tHV) and Total Analytical Hazard Value (taHV) are explained to emphasize the importance of minimizing environmental impact during analysis [23]. tHV refers to a measure that combines the toxicity of chemicals along with their potential environmental exposure factors, such as biodegradability. It is used to quantify the overall risk posed by a chemical substance. taHV is an extended version of the tHV that includes an additional parameter for volatility, which accounts for the analyst’s exposure to chemicals. This makes it especially relevant for assessing the hazards during analytical procedures in laboratories. Novel green sampling approaches are introduced through literature examples of environmentally friendly methods, including flow-through solid phase spectrometry [24], microextractions in bioanalysis [25], and the use of nanoparticles in sampling [26]. The future prospects and possibilities for green sampling are also explored.

The lecture then transitions to the direct analysis of samples, covering techniques such as remote sensing, process monitoring, and at-line non-destructive measurements [11]. Next, students are introduced to green sample preparation techniques. Guidelines for solvent selection are presented [27], with solvents categorized as preferred, usable, or undesirable [28]. Techniques such as head-space separation and microdistillation are discussed in the context of sample preparation utilizing gas-phase. For liquid-phase extractions, the unit briefly covers solid–liquid, liquid–liquid, sub- or supercritical water extraction, supercritical fluid extraction, and extraction using ionic liquids. Solid-phase extraction and microextraction are also mentioned, highlighting their green advantages.

The unit continues with a discussion of the green advantages and disadvantages of various analytical techniques familiar to the students, including capillary electrophoresis, chromatography, liquid chromatography, spectroscopy, and mass spectrometry. Special attention is given to the benefits and green prospects of supercritical fluid chromatography.

In the second part of the unit, students are engaged in critical reading of a paper on a low-cost palmtop capillary electrophoresis bioanalyzer [29]. The class, guided by the instructor, discusses the purpose of such a device, its connection to green analytical practices, and the aspects of capillary electrophoresis that can be miniaturized, including sample introduction. Students also explore the environmental impact of dichloromethane [30], discussing how this solvent can be safely replaced without compromising analytical efficiency [31]. Lastly, the development of Direct Analysis in Real-Time (DART) mass spectrometry is discussed [28], with particular emphasis on its benefits and importance in the context of green analytical chemistry practices.

*Unit 4: Greening strategies in analytical chemistry* has students exploring various approaches to “greening” current analytical methods and practices, with a particular emphasis on energy conservation. The discussion is supported by Nowak et al.’s publication [32], which features the carbon footprint associated with common analytical techniques, providing students with a clearer understanding of electricity consumption in these methods.

To deepen this understanding, students also examine online resources detailing strategies for energy savings in laboratories [33]. The potential of alternative energy sources is explored, including the use of microwave heating instead of conventional thermal methods [34] and the application of ultrasound in sample preparation [35].

Additionally, the unit covers the use of alternative solvents [36]. The concept of miniaturization as a strategy for green chemistry is introduced, particularly through the discussion of microextraction techniques [37]. As a practical example of successful miniaturization, the lab-on-a-chip technology is presented, illustrating how compact, integrated systems can contribute to more sustainable analytical practices [38].

*Unit 5 first part: Applications in green analytical chemistry* introduces students to modern green analytical chemistry techniques, focusing on their practical applications across various fields. A key topic is green bioanalysis, where the discussion centered on analyte extraction techniques, achieved recoveries, and detection limits of selected environmentally friendly methods [39].

In this unit, students also explore the green aspects of infrared spectroscopy, particularly its application in biodiagnostics [40]. Further exploration of environmental and industrial applications is guided by sections from the *Handbook of Green Analytical Chemistry* [11], which provided comprehensive insights into sustainable practices in these sectors. At the conclusion of this section, students engaged in an in-class activity where they read and discussed a review on switchable solvents and their applications [15]. A description of this task is provided in Supplementary File 1.

## Assessment and evaluation

*Unit 5 s part: Student assessment.* In this assessment, pairs of students are assigned with a current analytical method from the European Pharmacopoeia 11.0 [41]. Their task is to critically evaluate the method’s environmental impact using the AGREE, GAPI, and NEMI greenness assessment tools. After conducting the initial evaluation, students propose improvements to enhance the method’s green chemistry profile. The modified, greener method was then reassessed using the same tools and compared to the original method.

*Unit 6: Student assessment presentation.* Students present their findings via a PowerPoint presentation to the entire class, with each presentation lasting approximately 20 min. Their grades are primarily based on the originality and effectiveness of their proposed improvements, demonstrating their grasp of the course material.

The methods assigned included the analysis of the following compounds from the Pharmacopoeia:

- Etanercept (Etanerceptum)
- Follitropin concentrated solution
- Human coagulation factor VIIa (rDNA) concentrated solution
- Infliximab concentrated solution
- Teriparatide
- Terlipressin
- Insulin aspart
- Insulin glargine
- Filgrastim concentrated solution
- Glucagon, human

These methods, focusing on the analysis of proteins and peptides, are deliberately selected as the students were concurrently enrolled in a proteomics course, allowing for a more integrated learning experience. An example presentation is included in the supplementary material.

To ensure alignment between learning outcomes and assessments, detailed procedures are developed for each project and presentation. The grading criteria are designed to assess not only the technical proficiency of students but also their ability to apply GAC principles innovatively. Table 1S (supplementary material) outlines the criteria for evaluating the environmental impact analysis and the effectiveness of the proposed green chemistry improvements. The grading system followed the Swiss academic grading scale [42], with grades rounded to the nearest 0.5.

To provide a clearer representation of how the assignment supported the achievement of its learning objectives, we have summarized the performance of students in the main evaluation categories. Table 2 presents the distribution of students in each performance category: exceeds expectations (scores of 6), meets expectations (score of 4–5), and below expectations (scores of 1–3). These data illustrate the degree to which students met or surpassed the expected standards, highlighting areas of strength and identifying potential opportunities for improvement.

**Table 2:** Student performance

	Exceed expectations [%]	Meet expectations [%]	Below expectations [%]
Method analysis	33	59	8
Application of GAC principles	17	83	0
Clarity and organization	33	67	0
Originality and creativity	17	83	0
Overall impact	17	83	0

*Course evaluation:* Students provided anonymous feedback on the course, with overall impressions being largely positive. Notable comments included the following:

- Student 1: “Important and necessary.”
- Student 2: “The mix of practical exercises and theory helps us better assimilate the material.”
- Student 3: “Liked the group work.”
- Student 4: “The methods used to determine the greenness of an analytical method were interesting to discover.”

This feedback underscores the value of combining theoretical concepts with practical applications and collaborative learning.

## Outcomes and future directions

Based on student feedback and ongoing advancements in the field of GAC, several areas for potential course improvement have been identified. First, while students appreciated the balance between theoretical concepts and practical exercises, some suggested incorporating more hands-on activities related to real-world applications of green methods. Expanding laboratory sessions or integrating virtual lab simulations [43] could enhance students’ experiential learning and provide a more comprehensive understanding of green analytical techniques. Additionally, considering the rapid evolution of greenness assessment tools, updating the course content to include the latest methodologies and software for evaluating the environmental impact of analytical methods would ensure that students remain at the forefront of sustainable practices. Lastly, incorporating more interdisciplinary perspectives—such as the role of regulation in promoting green chemistry—could broaden students’ understanding of the public implications of their work.

To further embed green chemistry principles across the analytical chemistry curriculum, the integration of sustainability concepts in other related courses could be beneficial. For example, in courses on chromatography or spectroscopy, sections could be included that specifically focus on the development of greener methodologies, such as using eco-friendly solvents or energy-efficient instrumentation. Additionally, green chemistry could be emphasized in the design of student research projects, encouraging students to apply sustainable practices in their experimental designs. At the University of Applied Sciences Western Switzerland (HES-SO), students majoring in analytical chemistry are asked to identify how their bachelor thesis contributes to at least two of the United Nations (UN) Sustainable Development Goals (SDGs) [44]. By expanding the curriculum in this way, students will gain a more holistic and integrated understanding of green chemistry, preparing them to promote sustainability within the broader field of analytical science.

## Conclusions

This course on green analytical chemistry was designed to introduce undergraduate students to the principles and practices of sustainability in chemical analysis, with a focus on the application of green chemistry concepts across various analytical techniques. The curriculum was structured to balance theoretical knowledge with practical applications, ensuring that students not only understand the core principles but also develop hands-on experience in evaluating and improving the environmental impact of analytical methods. The integration of greenness assessment tools such as AGREE, GAPI, and NEMI allow students to engage in critical analysis and propose innovative solutions for enhancing the sustainability of established methods.

The course design emphasized active learning through collaborative exercises, case studies, and publication discussions, culminating in student presentations that showcased their ability to apply green principles in real-world contexts. The deliberate selection of methods related to protein and peptide analysis aligned the course content with the students’ concurrent studies, providing an interdisciplinary and cohesive learning experience.

Student feedback indicated that the course successfully blended theoretical concepts with collaborative work, fostering a deeper understanding of green analytical chemistry. However, areas for improvement were identified, such as incorporating hands-on laboratory work and updating course materials with the latest tools and developments in the field. Expanding green chemistry principles into other areas of the analytical chemistry curriculum would further reinforce the importance of sustainability in chemical education. Overall, the course has had a positive impact on students’ knowledge, skills, and awareness, preparing them to contribute to the growing demand for sustainable practices in the scientific community.

The positive outcomes of this course, as reflected in student feedback and performance data, underscore the effectiveness of integrating GAC principles into the undergraduate curriculum. Students not only demonstrated a strong grasp of GAC concepts but also showed an increased capacity for critical thinking and improvement in their approach to chemical analysis. These results confirm the course’s success in achieving its primary objective: to equip students with the knowledge and skills necessary to lead sustainable practices in their future careers.

## Supplementary Information

Below is the link to the electronic supplementary material.

Supplementary file1 (DOCX 21.7 KB)

Supplementary file2 (PDF 3.91 MB)
