# Supplementary material for: Green analytical chemistry: integrating sustainability into undergraduate education
Source: Anal Bioanal Chem. 2024 Dec 6;417(4):665–73. doi: 10.1007/s00216-024-05680-4 (PMC11772533; doi:10.1007/s00216-024-05680-4)
Supplement: Supplementary file 1 — Supplementary file1 (DOCX 21.7 KB) [file 216_2024_5680_MOESM1_ESM.docx]

# Supplementary Information

**Instructions for Jigsaw Collaborative Learning Activity on Switchable Solvents Review**

At the conclusion of the first part of the Unit 5, students engaged in an in-class activity where they read and discussed a review on switchable solvents and their applications [1]. The instructions used to perform this task are described below:

1. *Form Initial Groups*
   Divide into three groups. Each group will be assigned a specific section of the paper titled *"A Comprehensive Review on Recent Developments and Future Perspectives of Switchable Solvents and Their Applications in Sample Preparation Techniques."*

Group 1: Read from the beginning of the paper up to Chapter 5.1.

Group 2: Read from Chapter 5.1 to Chapter 5.10.

Group 3: Read from Chapter 5.10 to the end of the paper.

1. *Read and Digest Material*
   Spend the next 45 minutes reading your assigned section carefully. Focus on understanding the key points, findings, and implications within your section. Take notes as needed to help you summarize the material.
2. *Form Mixed Groups*
   After the reading phase, form new groups that include one member from each of the original reading groups. Each new group should have a representative who read each section of the paper.
3. *Share and Discuss*
   In your mixed groups, each member will take turns summarizing their assigned section of the paper. In another 45 minutes students explain the main ideas, findings, and any important details, ensuring that all group members understand each part of the review.
4. *Instructor’s Role*
   The instructor will be available to help facilitate your discussions. If you have questions or need clarification, raise them with the instructor as they circulate among groups. Use this time to dive deeper into the material and explore complex concepts together.
5. *Objective*This activity aims to build a comprehensive understanding of the entire review paper through collaborative learning. Focus on clear communication to help your peers understand your section, and engage actively to grasp insights from other sections.

These instructions are intended as a flexible guide. Instructors may adapt the activity based on the class dynamics, students' knowledge level, or other contextual factors to best suit the needs and engagement of the students. Adjustments to timing, group composition, or discussion depth can be made as appropriate.

**Evaluation of the students’ presentation example from Supplementary File 2**

The students' presentation on evaluating the greenness of the current analytical method on Follitropin using Isoelectric Focusing Electrophoresis and High-Performance Liquid Chromatography (HPLC) was detailed and well-articulated, demonstrating a solid understanding of the application of greenness tools. They effectively highlighted the limitations of the current method and presented a feasible case for improvement. The introduction of miniaturization and the use of Ultra High-Performance Liquid Chromatography (UHPLC) as a replacement for HPLC were intuitive recommendations that showcased an approach to reducing environmental impact. These proposed changes resulted in better greenness scores, as quantified by the evaluation tools, emphasizing their potential to make the method more sustainable. The students effectively connected the technical improvement possibilities with environmental benefits.

Table 1S. Assessment criteria

| **Grade** | **6 - Excellent** | **5 - Good** | **4 - Sufficient** | **≤3 - Poor** |
| --- | --- | --- | --- | --- |
| **Method Analysis** | Thorough and accurate analysis of the analytical method using greenness assessment tools. All data are clearly presented and fully support conclusions. | Analysis is mostly accurate with minor errors. Data support most conclusions, but some gaps or unclear points exist. | Significant errors or omissions in analysis. Data partially support conclusions, but some conclusions lack evidence. | Inaccurate or incomplete analysis. Data do not support conclusions or are largely missing. |
| **Application of GAC Principles** | Innovative and comprehensive improvements proposed, fully aligning with green chemistry principles. | Improvements proposed align well with green chemistry principles but lack depth or originality. | Improvements proposed are limited or partially aligned with green chemistry principles. | Improvements are either missing or poorly aligned with green chemistry principles. |
| **Clarity and Organization** | Presentation is well-organized with a logical flow. Visual aids are clear, relevant, and effectively enhance understanding. | Presentation is organized, though some points may be unclear. Visual aids are mostly relevant but may not fully enhance understanding. | Presentation lacks clear organization, making it difficult to follow. Visual aids are minimally effective or irrelevant. | Presentation is poorly organized and difficult to follow. Visual aids are missing or do not contribute to understanding. |
| **Originality and Creativity** | Presentation offers highly original and creative solutions or perspectives. | Presentation includes some original ideas or creative solutions. | Presentation offers few original ideas or creative solutions. | Presentation lacks originality or creativity. Shows little to no innovative thinking. |
| **Overall Impact** | Presentation is highly effective in conveying key ideas. | Presentation is effective but could be more engaging or impactful. | Presentation has some impact but key ideas are not fully conveyed. | Presentation fails to effectively communicate the key ideas. |

1. Ullah N, Tuzen M (2023) A comprehensive review on recent developments and future perspectives of switchable solvents and their applications in sample preparation techniques. Green Chem 25:1729–1748. https://doi.org/10.1039/D3GC00020F
