# Supplementary material for: Green analytical chemistry: integrating sustainability into undergraduate education
Source: Anal Bioanal Chem. 2024 Dec 6;417(4):665–73. doi: 10.1007/s00216-024-05680-4 (PMC11772533; doi:10.1007/s00216-024-05680-4)
Supplement: Supplementary file 2 — Supplementary file2 (PDF 3.91 MB) [file 216_2024_5680_MOESM2_ESM.pdf]

Hes·so// VALAIS WALLIS
Haute Ecole d'Ingénierie
Hochschule für Ingenieurwissenschaften

Optimisation of analytical methods about the Follitropin

| Green analytical chemistry

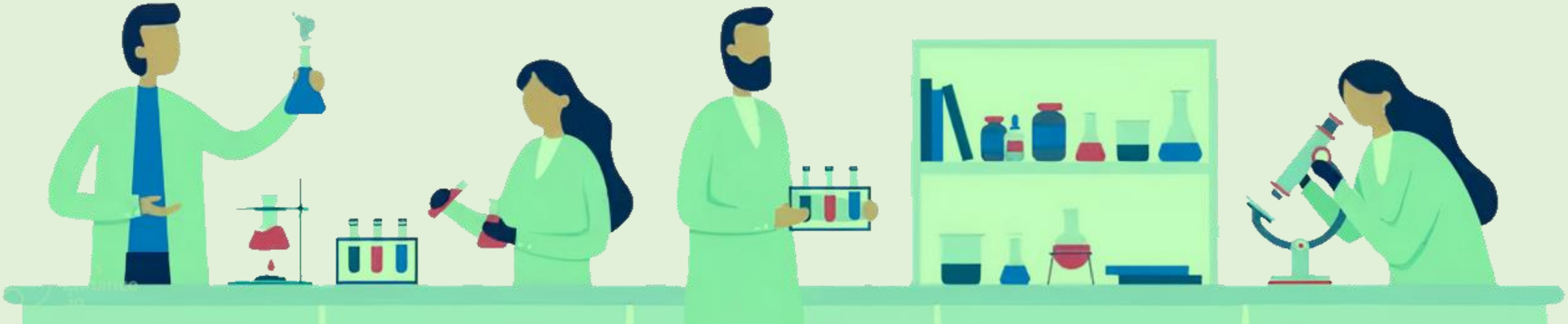

Hes·so // VALAIS WALLIS
Haute Ecole d'Ingénierie
Hochschule für Ingenieurwissenschaften

Follitropin

What is it ?

Produced in the **mammalian cells** by a method based on recombinant **DNA** technology

Heterodimeric glycoprotein

Structure of human Follicle-Stimulating Hormone (FSH)

2 subunits :

α-subunit
APDVQDCPEC TLQENPFFSQ PGAPILQCMG CCFSRAYPTP 40
LRSKKTMLVQ KNVTSESTCC VAKSYNRVTV MGGFKVENHT 80
ACHCSTCYYH KS 92

β-subunit
NSCELTNITI AIEKEECRFC ISINTTWCAG YCYTRDLVYK 40'
DPARPKIQKT CTFKELVYET VRVPGCAHHA DSLYTYPVAT 80'
QCHCGKCDSD STDCTVRGLG PSYCSFGEMK E 111'

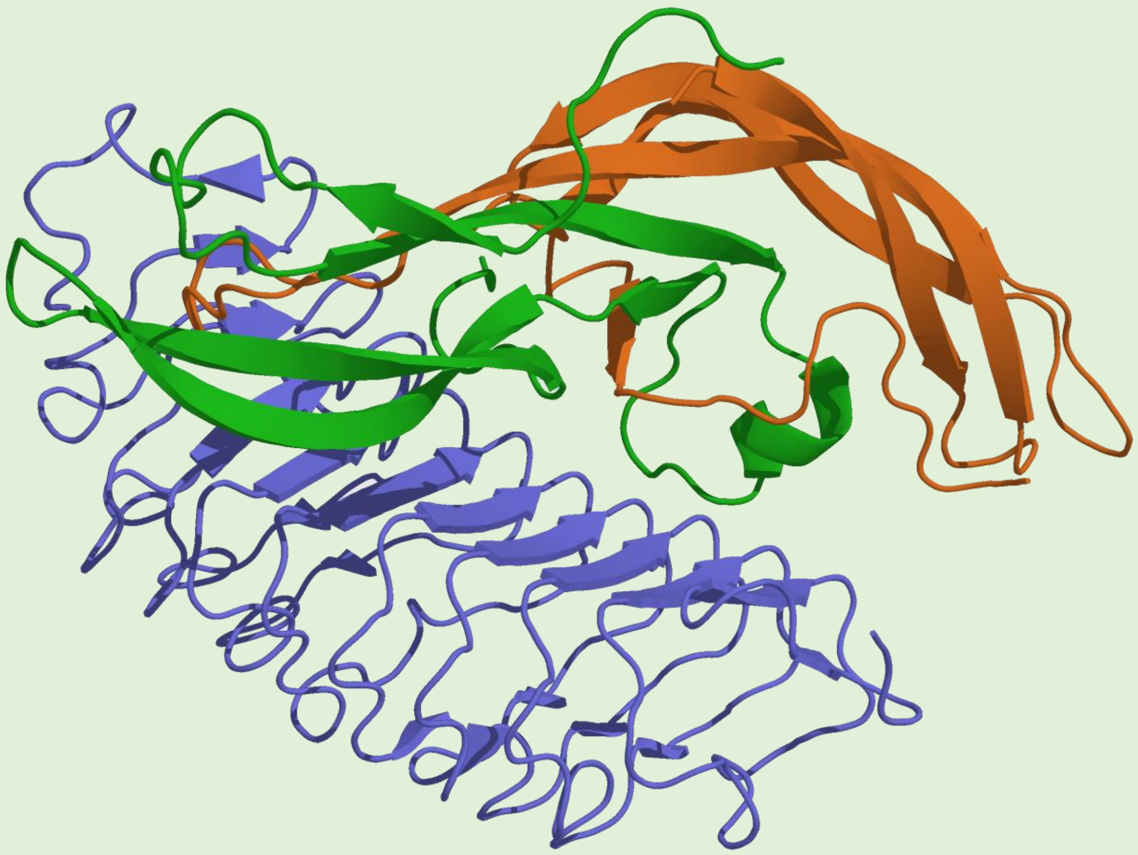

Identification
IEF

Identification

IEF

Sample

1. Desalt and concentrate
2. Reconstitue the recovered material in water

Other solutions

1. Reference solution : Follitropin CRS vial
2. pH gradient : ampholyte and electrode buffer
3. Catholyte : Glycine R
4. Anolyte : Aspartic and glutamic acid

IEF

AGREE

Criterion	Description	Weight	The analysis
1	Sampling procedure	2	Off-line analysis
2	Sample amount	2	7
3	Analytical device	1	Off-line
4	Step in the sample prep.	2	3 or fewer
5	Degree of automation	2	Semi-antomatic
	Sample preparation		Not miniaturized
6	Derivatization agent	2	none
7	Amount of waste	3	5
8	No of analyte in a single run	2	2
	Sample throughput		4
9	Power consumption [kWh]	2	0.066
10	Type of reagent	2	None-reagent are from bio-based source
11	Toxic reagent ?	2	no
12	Threath	2	Corrosive

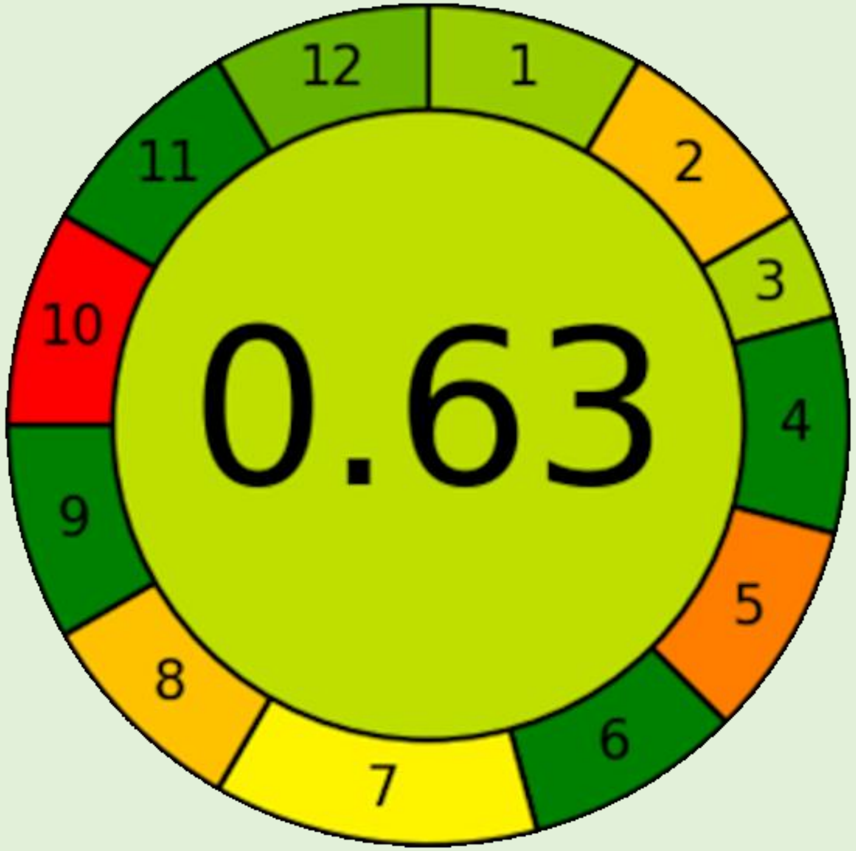

IEF

GAPI

Quantification
Procedure only for qualification

Sample preparation
Off-line
Chemical or physical
None
Under normal conditions
Simple procedures
Macro-extraction
Solvent-free methods
Simple treatments

Reagents and solvents
< 10 mL (< 10 g)
Slightly toxic, slight irritant; NFPA health hazard score of 0 or 1. No special hazards.
Highest NFPA flammability or instability score of 0 or 1. No special hazards.

Instrumentation
<= 0.1 kWh per sample
Hermetic sealing of the analytical process
1-10 mL (1-10 g)
No treatment

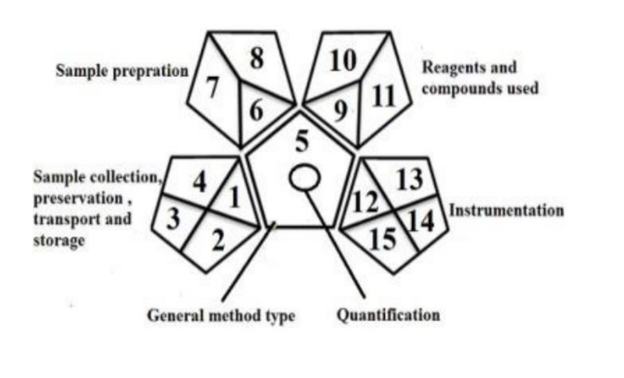

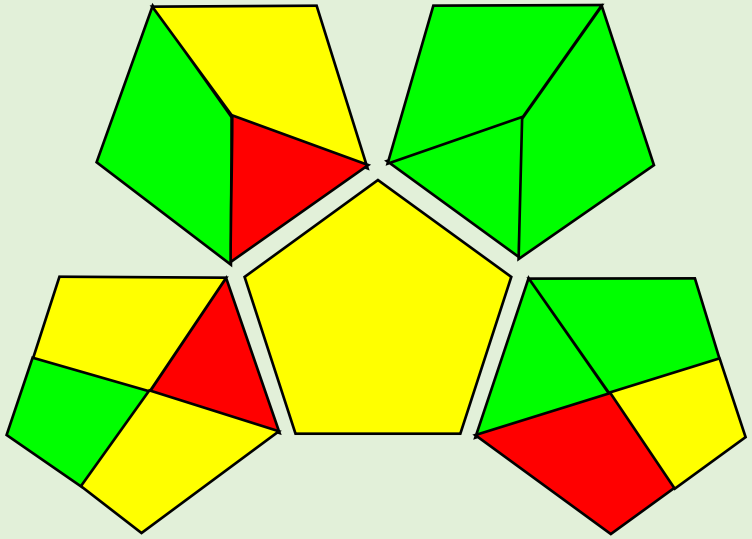

IEF

Concentration and desalting

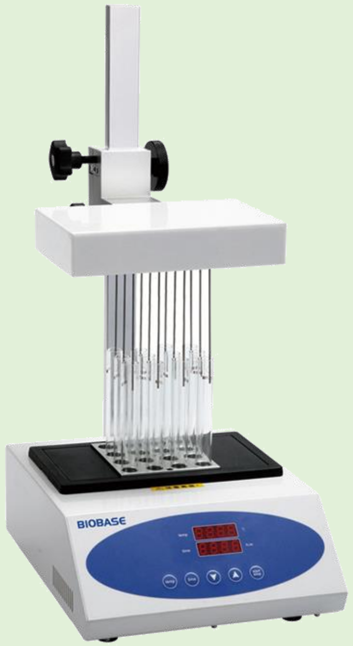

Nitrogen evaporator

(Nitrogen flux + heating)

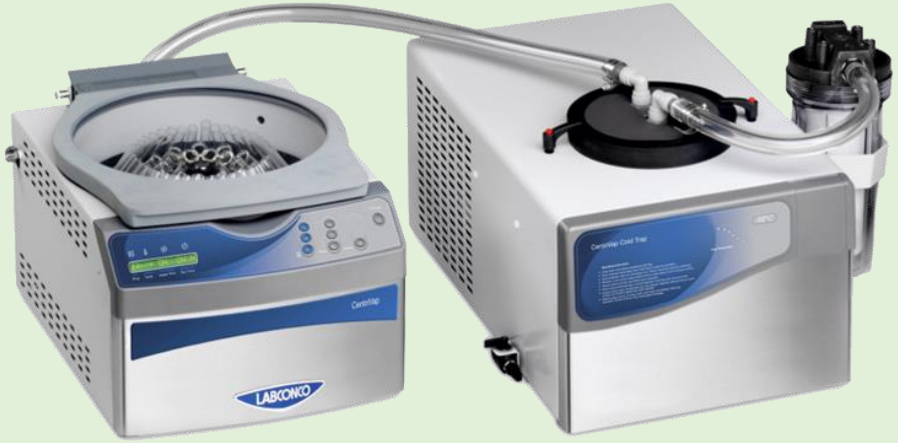

Centrivap solvent system

(centrifugation + low pressure)

IEF

Concentration and desalting

SPE :

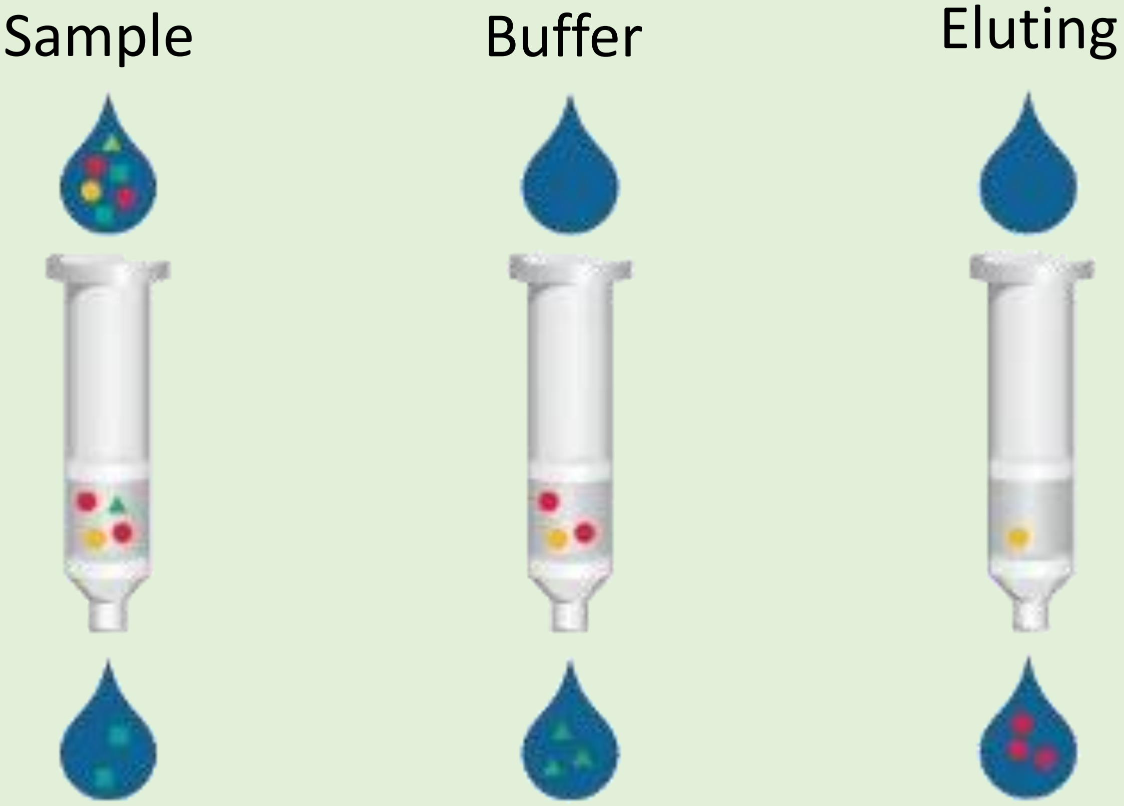

IEF

Miniaturizing

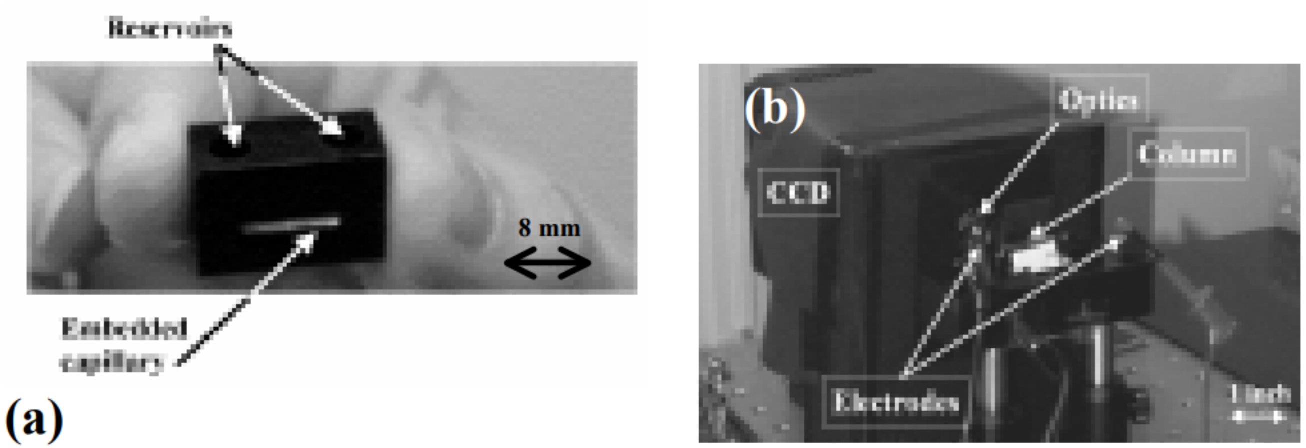

Figure 2. Miniaturized IEF System. (a) Capillary cartridge and **(b)** the hardware mount for the excitation source and optics, the cartridge mount, the emission filtering/focusing optics, and the CCD detection component of the diagnostic are shown.

MINIATURIZED CAPILLARY ISOELECTRIC FOCUSING (cIEF): TOWARDS A PORTABLE HIGH-SPEED SEPARATION METHOD

IEF

AGREE

Criterion	Description	Weight	The analysis
1	Sampling procedure	2	Off-line analysis
2	Sample amount	2	2
3	Analytical device	1	Off-line
4	Step in the sample prep.	2	3 or fewer
5	Degree of automation	2	Semi-antomatic
	Sample preparation		Miniaturized
6	Derivatization agent	2	none
7	Amount of waste	3	3
8	No of analyte in a single run	2	2
	Sample throughput		30
9	Power consumption [kWh]	2	<0.001 kWh
10	Type of reagent	2	Some of the reagent are bio-based
11	Toxic reagent ?	2	no
12	Threath	2	Corrosive

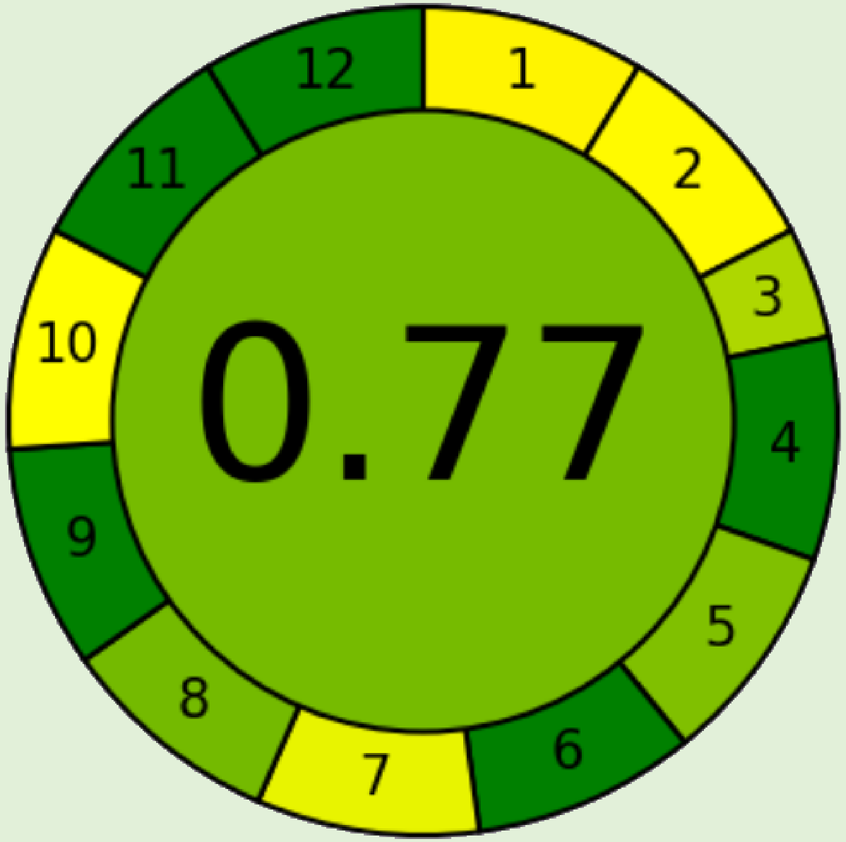

IEF

GAPI

Quantification
Procedure only for qualification

Sample preparation
Off-line
Chemical or physical
None
Under normal conditions
Simple procedures
Micro-extraction
Solvent-free methods
Simple treatments

Reagents and solvents
< 10 mL (< 10 g)
Slightly toxic, slight irritant; NFPA health hazard score of 0 or 1. No special hazards.
Highest NFPA flammability or instability score of 0 or 1. No special hazards.

Instrumentation
<= 0.1 kWh per sample
Hermetic sealing of the analytical process
1-10 mL (1-10 g)
Recycling

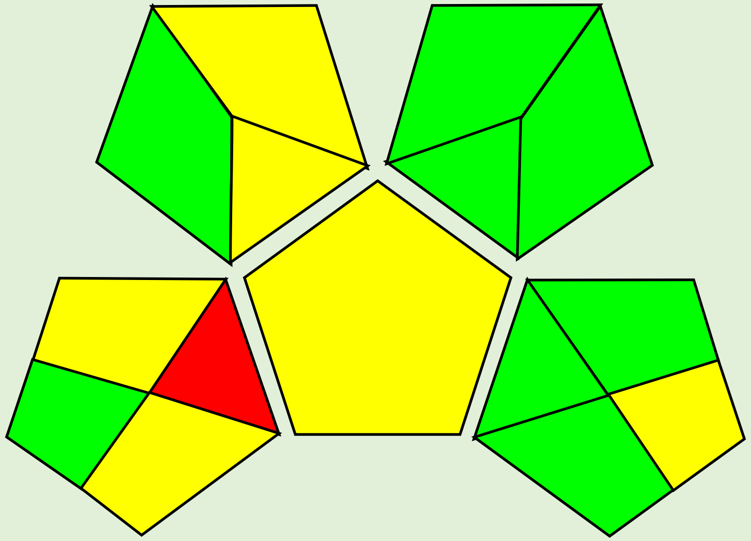

Separation of the α– and β-subunits HPLC

HPLC

AGREE

Criterion	Description	Weight	The analysis
1	Sampling procedure	2	Off-line analysis
2	Sample amount	2	1 ml
3	Analytical device	1	On-line
4	Step in the sample prep.	2	3 or fewer
5	Degree of automation	2	Semi-automatic
	Sample preparation		Not miniaturized
6	Derivatization agent	2	none
7	Amount of waste	3	57 ml
8	No of analyte in a single run	2	2
	Sample throughput		1.05/h
9	Power consumption [kWh]	2	1
10	Type of reagent	2	None-reagent are from bio-based source
11	Toxic reagent ?	2	Yes
		2	15 ml/run
12	Threath	2	Highly flammable

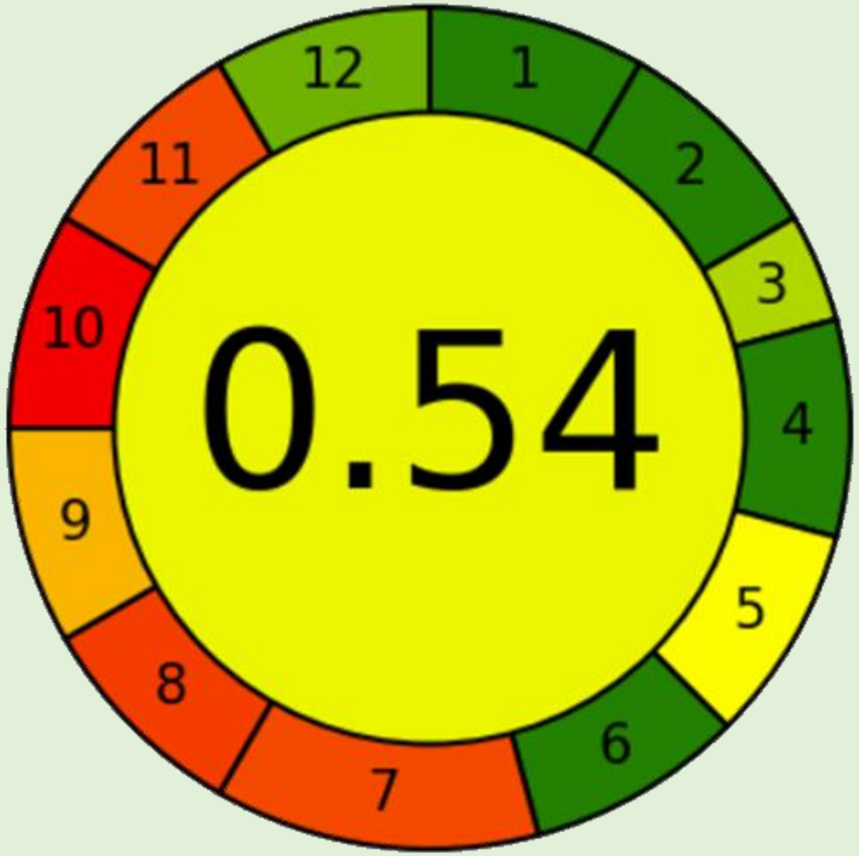

HPLC

GAPI

Quantification
Procedure only for qualification

Sample preparation
On-line or at-line
Chemical or physical
None
Under normal conditions
Simple procedures
Scale of extraction (6)
Non-green solvents / reagents
None

Reagents and solvents
10-100 mL (10-100 g)
Moderately toxic; could cause temporary incapacitation; NFPA = 2 or 3.
Highest NFPA flammability or instability score = 2 or 3, or a special hazard is used.

Instrumentation
<= 1.5 kWh per sample
Hermetic sealing of the analytical process
> 10 mL (>10 g)
No treatment

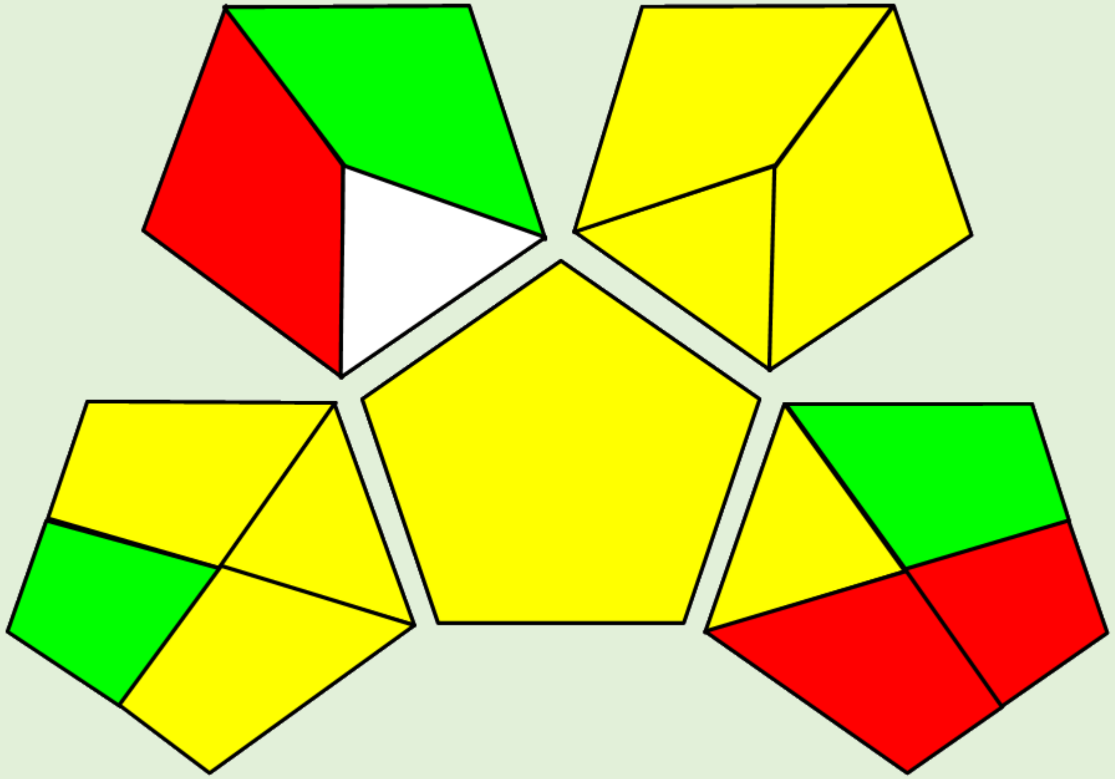

HPLC

Transfer to U-HPLC

Current Column	
Length (mm):	250
Diameter (mm):	4.6
Particle Size (µm):	5.0
**Peak Details (Critical Pair)**	
Actual R_S (Resolution Factor):	2.00
**Current Method Conditions**	
Flow (mL/min):	1.000
Injection Volume (µL):	800.0
Max Observed Pressure:	80
Pressure Units:	bar

Planned Column		
Length (mm):	100	
Diameter (mm):	2.1	
Particle Size (µm):	2.2	
**Peak Details (Critical Pair)**		
Predicted R_S Change Factor:	0.95	(-4.7%)
Predicted R_S:	1.91	Baseline resolution achieved
**Recommended Method Conditions**		
Boost Factor:	1.00	

* Use this factor to increase the flow rate of the fast LC method. Note: if factor other than 1 is used, the resolution calculation is disabled.

☐ Adjust Flow

Flow (mL/min):	0.474
Injection Volume (µL):	69.9
Estimated Max Observed Pressure:	376 bar

HPLC

Transfer to U-HPLC

Current Gradient Table

Step	Time (min)	%A	%B	%C	%D
1	0.00	100.0	0.0	0.0	0.0
2	2.00	100.0	0.0		
3	8.00	76.0	24.0		
4	17.00	76.0	24.0		
5	36.00	70.0	30.0		
6	41.00	25.0	75.0		
7	46.00	25.0	75.0		
8	47.00	100.0	0.0		
9	57.00	100.0	0.0		
10					

End Time: 57.000

Recommended Reconditioning Time: 16.204

Planned Gradient Table

Step	Time (min)	%A	%B	%C	%D
1	0.00	100.0	0.0	0.0	0.0
2	0.35	100.0	0.0	0.0	[illegible]
3	1.41	76.0	24.0	0.0	[illegible]
4	2.99	76.0	24.0	0.0	[illegible]
5	6.34	70.0	30.0	0.0	[illegible]
6	7.22	25.0	75.0	0.0	[illegible]
7	8.10	25.0	75.0	0.0	[illegible]
8	8.27	100.0	0.0	0.0	[illegible]
9	10.03	100.0	0.0	0.0	[illegible]
10					[illegible]

End Time: 10.032

Recommended Reconditioning Time: 2.852

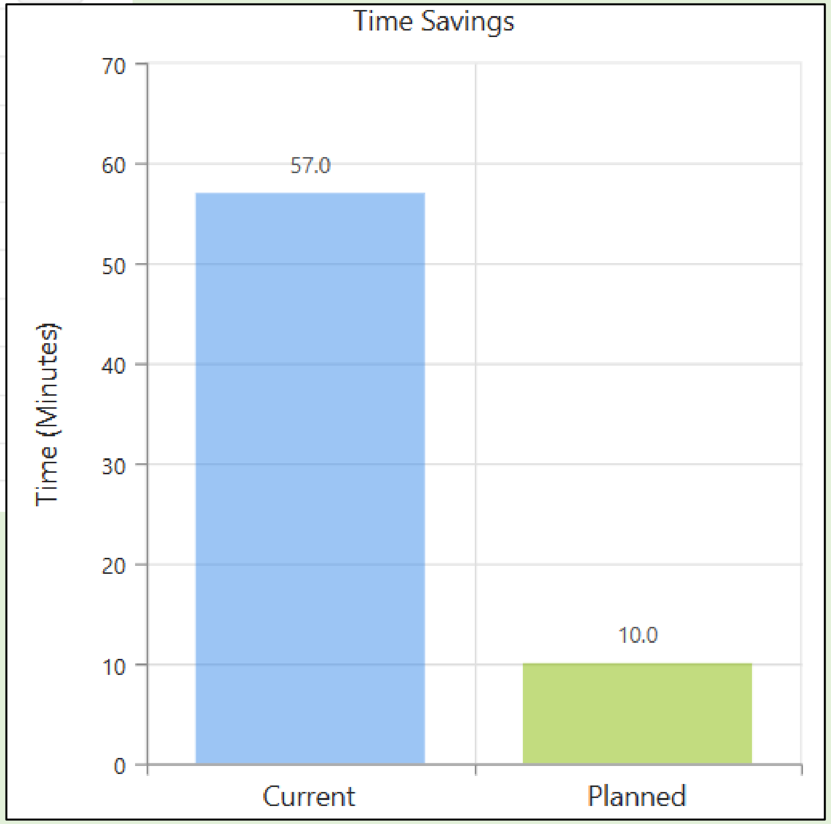

HPLC

AGREE

Criterion	Description	Weight	The Green analysis
1	Sampling procedure	2	Off-line analysis
2	Sample amount	2	1 ml
3	Analytical device	1	On-line
4	Step in the sample prep.	2	3 or fewer
5	Degree of automation	2	Semi-automatic
	Sample preparation		Not miniaturized
6	Derivatization agent	2	none
7	Amount of waste	3	4.7 ml
8	No of analyte in a single run	2	2
	Sample throughput		6/h
9	Power consumption [kWh]	2	0.1
10	Type of reagent	2	Some reagents are bio-based
11	Toxic reagent ?	2	Yes
		2	2 ml/run
12	Threath	2	Highly flammable

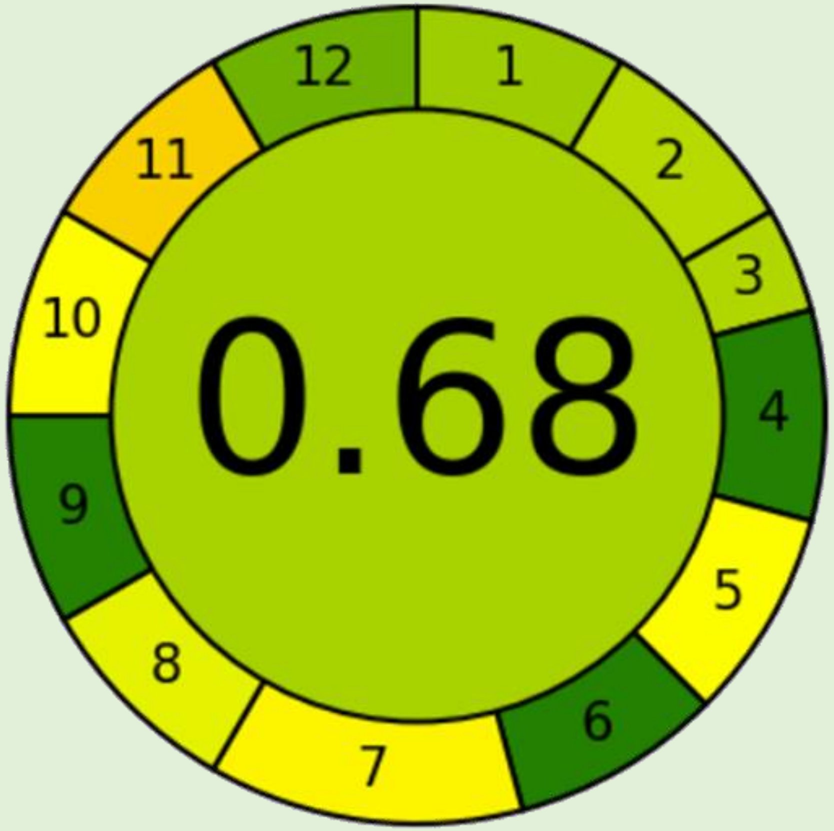

HPLC

GAPI

Quantification
Procedure only for qualification

Sample preparation
On-line or at-line
Chemical or physical
None
Under normal conditions
Simple procedures
Scale of extraction (6)
Green solvents / reagents
None

Reagents and solvents
< 10 mL (< 10 g)
Moderately toxic; could cause temporary incapacitation; NFPA = 2 or 3.
Highest NFPA flammability or instability score = 2 or 3, or a special hazard is used.

Instrumentation
<= 0.1 kWh per sample
Hermetic sealing of the analytical process
1-10 mL (1-10 g)
No treatment

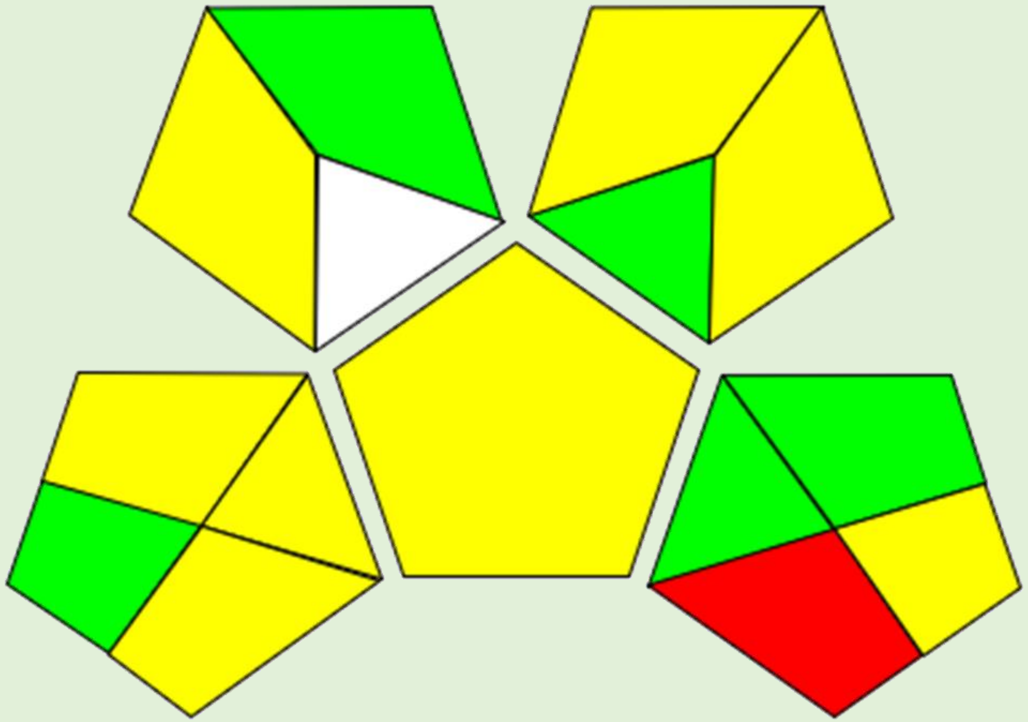

Glycan analysis
HPLC

HPLC

AGREE

Criterion	Description	Weight	The analysis
1	Sampling procedure	2	Off-line analysis
2	Sample amount	2	1 ml
3	Analytical device	1	On-line
4	Step in the sample prep.	2	7
5	Degree of automation	2	Semi-automatic
	Sample preparation		Not miniaturized
6	Derivatization agent	2	none
7	Amount of waste	3	47
8	No of analyte in a single run	2	5
	Sample throughput		0.5/h
9	Power consumption [kWh]	2	1.5
10	Type of reagent	2	None-reagent are from bio-based source
11	Toxic reagent ?	2	Yes
		2	15 ml/run
12	Threath	2	Highly flammable

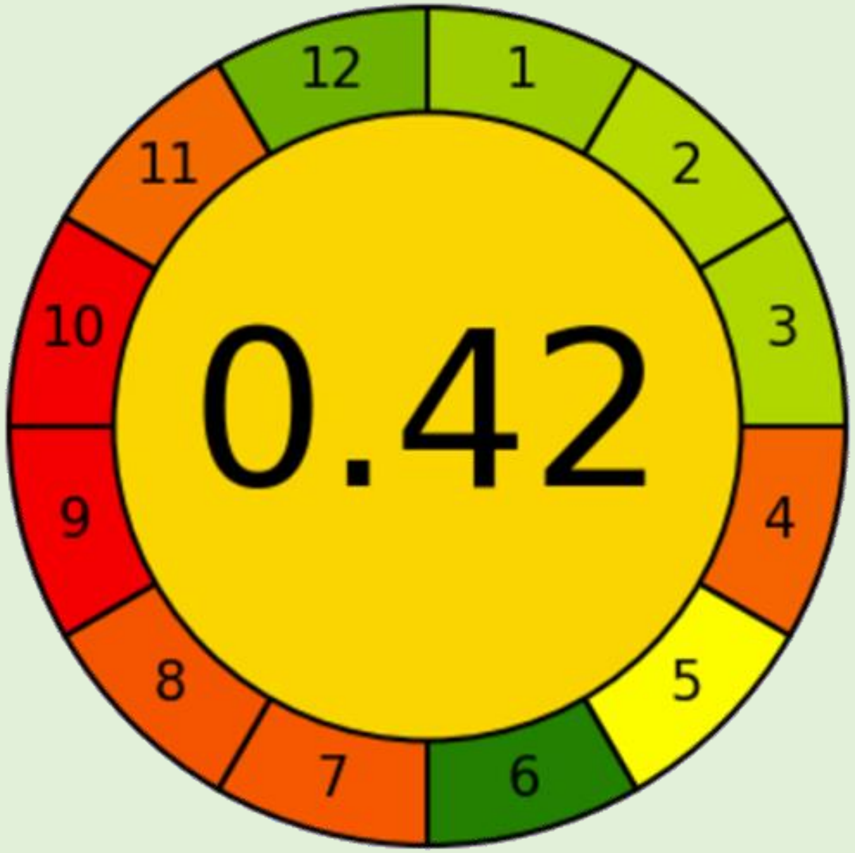

HPLC

GAPI

Quantification
Procedure only for qualification

Sample preparation
On-line or at-line
Chemical or physical
None
Under normal conditions
Extraction required
Micro-extraction
Non-green solvents / reagents
None

Reagents and solvents
10-100 mL (10-100 g)
Moderately toxic; could cause temporary incapacitation; NFPA = 2 or 3.
Highest NFPA flammability or instability score = 2 or 3, or a special hazard is used.

Instrumentation
<= 1.5 kWh per sample
Hermetic sealing of the analytical process
> 10 mL (>10 g)
No treatment

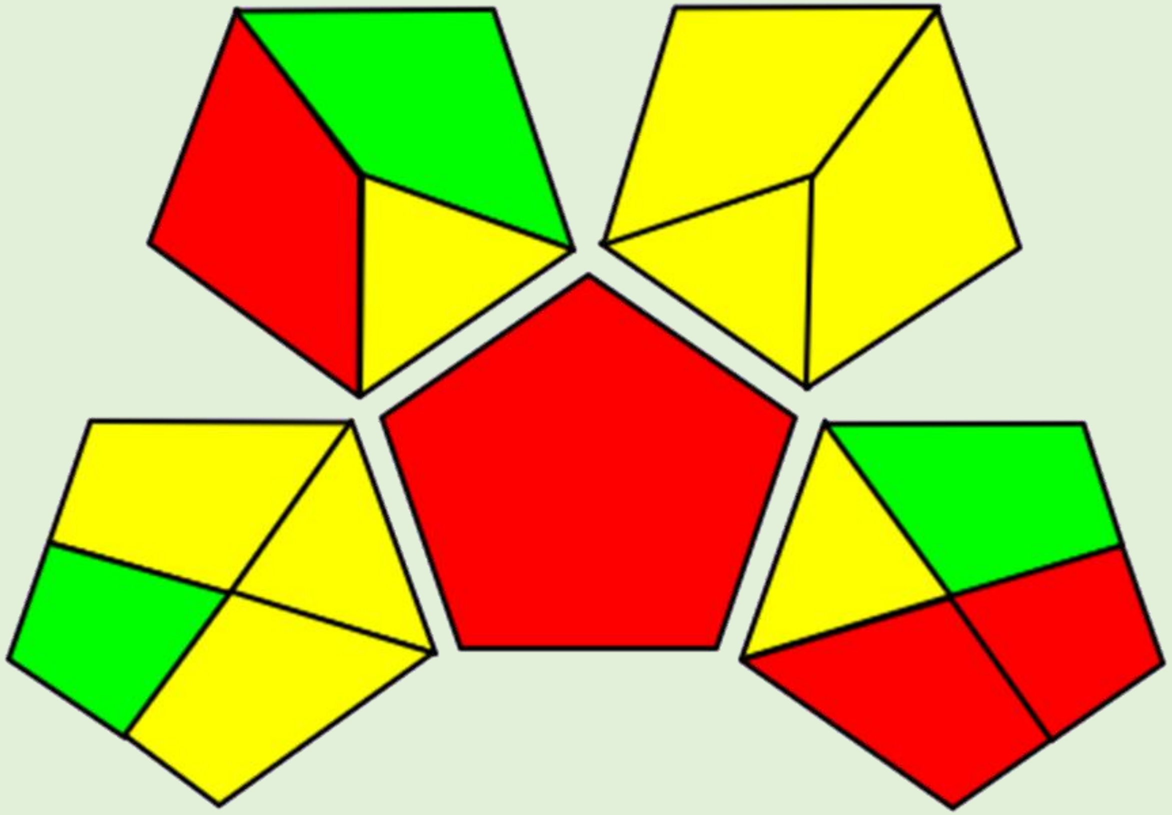

HPLC

Transfer to U-HPLC

Current Column

Length (mm):	75
Diameter (mm):	7.5
Particle Size (µm):	10.0

Peak Details (Critical Pair)

Actual R_S (Resolution Factor):	1.50

Current Method Conditions

Flow (mL/min):	0.400
Injection Volume (µL):	50.0
Max Observed Pressure:	40
Pressure Units:	bar

Planned Column

Length (mm):	50
Diameter (mm):	2.1
Particle Size (µm):	5.0

Peak Details (Critical Pair)

Predicted R_S Change Factor:	1.15	(15.5%)
Predicted R_S:	1.73	Baseline resolution achieved

Recommended Method Conditions

Boost Factor: 1.00

* Use this factor to increase the flow rate of the fast LC method. Note: if factor other than 1 is used, the resolution calculation is disabled.

☐ Adjust Flow

Flow (mL/min):	0.063
Injection Volume (µL):	2.3
Estimated Max Observed Pressure:	213 bar

HPLC

Transfer to U-HPLC

Current Gradient Table

Step	Time (min)	%A	%B	%C	%D
1	0.00	20.0	0.0	80.0	0.0
2	5.00	20.0	0.0	80.0	
3	21.00	20.0	4.0	76.0	
4	61.00	20.0	25.0	55.0	
5	62.00	20.0	50.0	30.0	
6	71.00	20.0	50.0	30.0	
7	72.00	20.0	0.0	80.0	
8	117.00	20.0	0.0	80.0	
9					
10					

End Time: 117.000

Recommended Reconditioning Time: 32.306

Planned Gradient Table

Step	Time (min)	%A	%B	%C	%D
1	0.00	20.0	0.0	80.0	0.0
2	1.67	20.0	0.0	80.0	0.0
3	7.00	20.0	4.0	76.0	[illegible]
4	20.33	20.0	25.0	55.0	[illegible]
5	20.67	20.0	50.0	30.0	[illegible]
6	23.67	20.0	50.0	30.0	[illegible]
7	24.00	20.0	0.0	80.0	[illegible]
8	39.00	20.0	0.0	80.0	[illegible]
9					[illegible]
10					[illegible]

End Time: 39.000

Recommended Reconditioning Time: 10.769

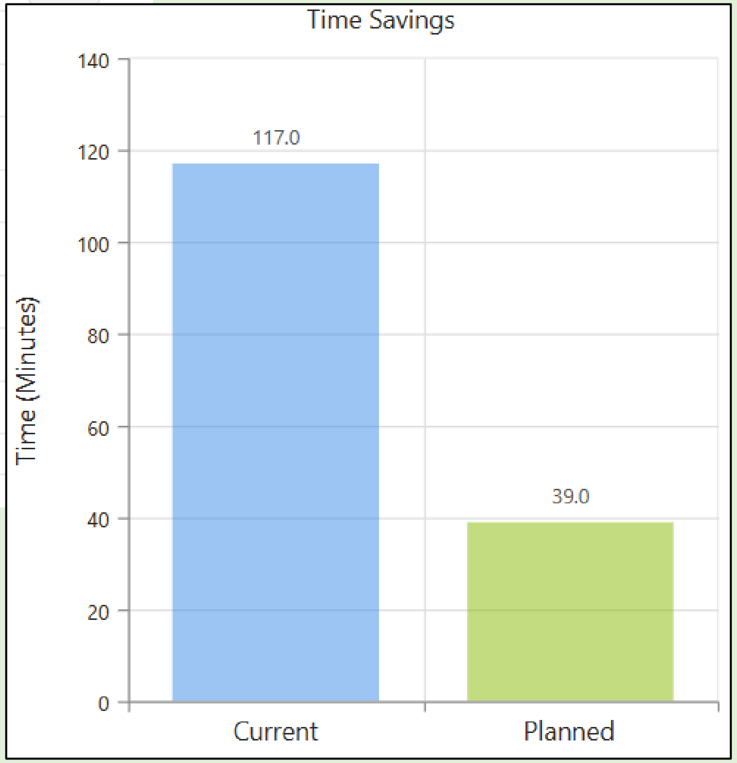

HPLC

AGREE

Criterion	Description	Weight	The Green analysis
1	Sampling procedure	2	Off-line analysis
2	Sample amount	2	1 ml
3	Analytical device	1	On-line
4	Step in the sample prep.	2	7
5	Degree of automation	2	Semi-automatic
	Sample preparation		Not miniaturized
6	Derivatization agent	2	none
7	Amount of waste	3	2.45
8	No of analyte in a single run	2	5
	Sample throughput		1.5/h
9	Power consumption [kWh]	2	0.1
10	Type of reagent	2	Some reagents are bio-based
11	Toxic reagent ?	2	Yes
		2	1 ml/run
12	Threath	2	Highly flammable

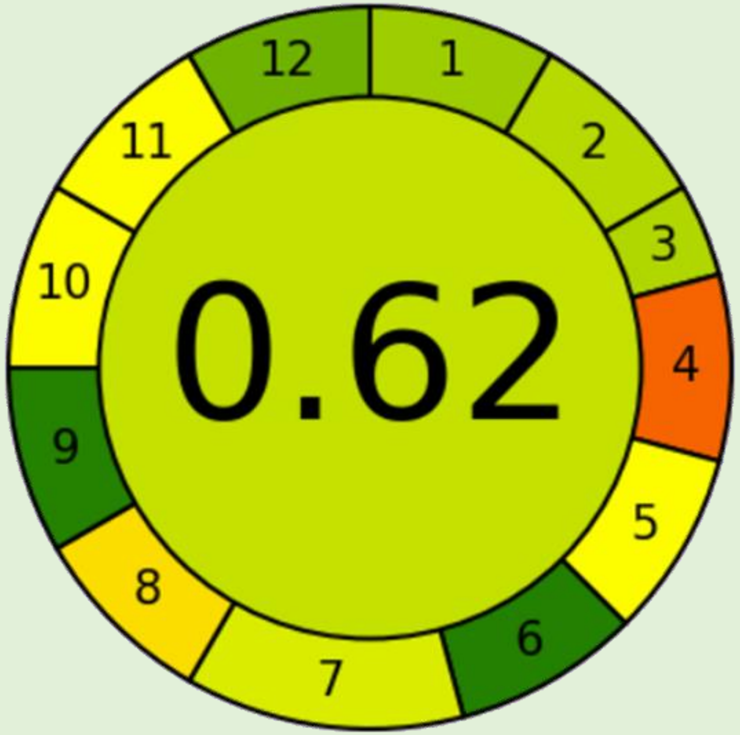

HPLC

GAPI

Quantification
Procedure only for qualification

Sample preparation
On-line or at-line
Chemical or physical
None
Under normal conditions
Extraction required
Micro-extraction
Green solvents / reagents
None

Reagents and solvents
< 10 mL (< 10 g)
Moderately toxic; could cause temporary incapacitation; NFPA = 2 or 3.
Highest NFPA flammability or instability score = 2 or 3, or a special hazard is used.

Instrumentation
<= 0.1 kWh per sample
Hermetic sealing of the analytical process
1-10 mL (1-10 g)
No treatment

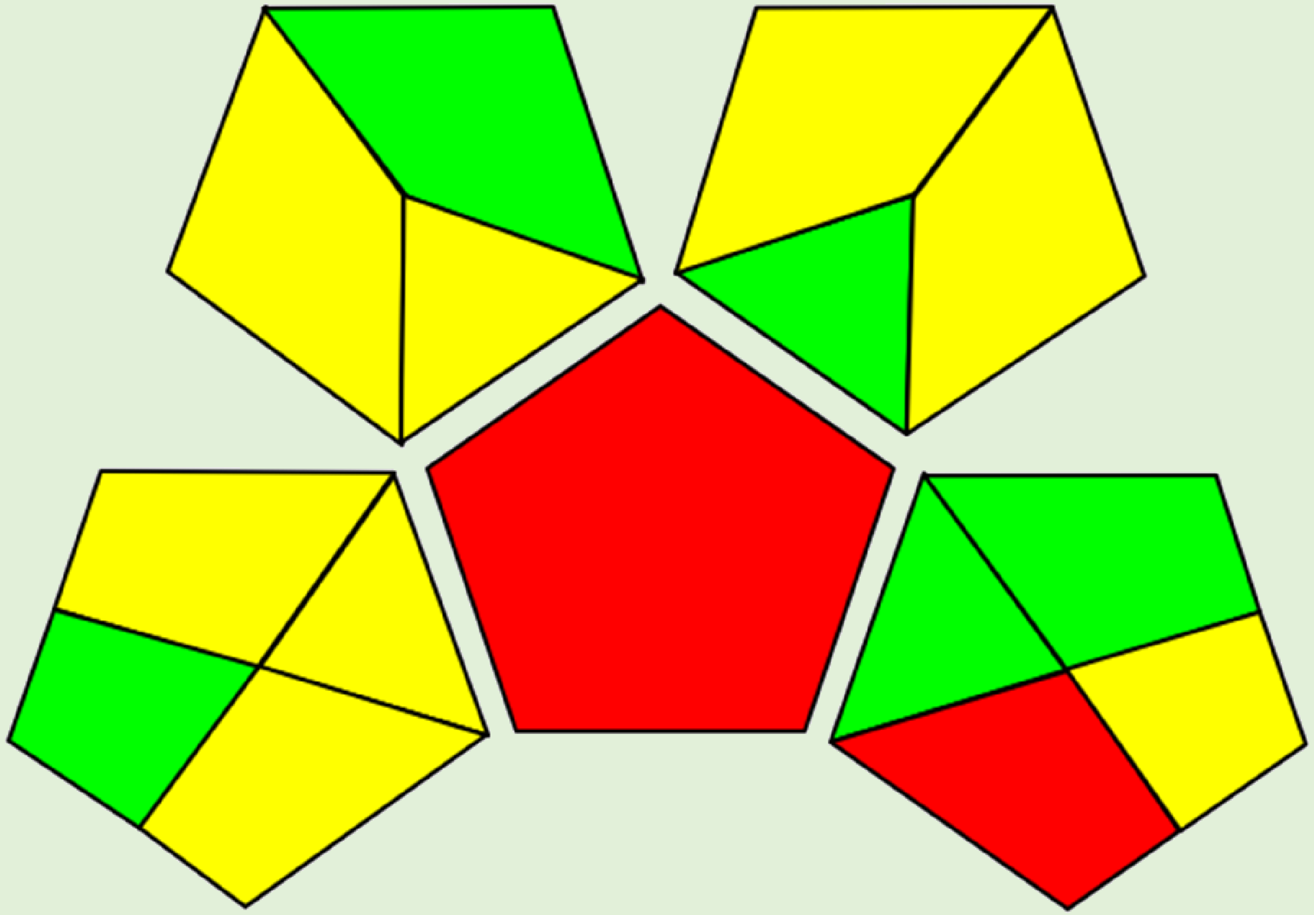

Conclusion

Conclusion

Thank you for your attention !

Do you have any questions ?
